# 

**Published:** 1881-11

**Authors:** 


					﻿Whole Number,
CCXXXII.
NOVEMBER, 1881.
Number 4,
Volume XXI.
THE
BUFFALO
Medical and Surgical Journal.
EDITORS:
THOS. I.OTHROP, M.
I’. W. VAN l’EYMA, M.
A. R. DAVIDSON, M.
Ll'CIEN HOWE, M. !>.,
HERMAN MVNTER, M. D.
BUFFALO :
Published by the Medical Journal Association.
Read the Notice of this Journal among the Advertisements.
Entered at the Post-Office at Buffalo, N. Y., as Second-Class Mail Matter.
				

